# Automated Detection and Removal of Cardiac and Pulse Interferences from Neonatal EEG Signals

**DOI:** 10.3390/s21196364

**Published:** 2021-09-23

**Authors:** Gabriella Tamburro, Pierpaolo Croce, Filippo Zappasodi, Silvia Comani

**Affiliations:** 1 Behavioral Imaging and Neural Dynamics Center, G. d’Annunzio University of Chieti–Pescara, 66100 Chieti, Italy; comani@unich.it; 2 Department of Neuroscience, Imaging and Clinical Sciences, G. d’Annunzio University of Chieti–Pescara, 66100 Chieti, Italy; pierpaolo.croce@unich.it (P.C.); filippo.zappasodi@unich.it (F.Z.); 3 Institute for Advanced Biomedical Technologies, G. d’Annunzio University of Chieti–Pescara, 66100 Chieti, Italy

**Keywords:** neonatal electroencephalography (EEG), automated artefact removal, independent component analysis (ICA), blind source separation methods (BSS), cardiac interference, pulse interference, electrocardiography (ECG)

## Abstract

Electrical cardiac and pulsatile interference is very difficult to remove from electroencephalographic (EEG) signals, especially if recorded in neonates, for which a small number of EEG channels is used. Several methods were proposed, including Blind Source Separation (BSS) methods that required the use of artificial cardiac-related signals to improve the separation of artefactual components. To optimize the separation of cardiac-related artefactual components, we propose a method based on Independent Component Analysis (ICA) that exploits specific features of the real electrocardiographic (ECG) signals that were simultaneously recorded with the neonatal EEG. A total of forty EEG segments from 19-channel neonatal EEG recordings with and without seizures were used to test and validate the performance of our method. We observed a significant reduction in the number of independent components (ICs) containing cardiac-related interferences, with a consequent improvement in the automated classification of the separated ICs. The comparison with the expert labeling of the ICs separately containing electrical cardiac and pulsatile interference led to an accuracy = 0.99, a false omission rate = 0.01 and a sensitivity = 0.93, outperforming existing methods. Furthermore, we verified that true brain activity was preserved in neonatal EEG signals reconstructed after the removal of artefactual ICs, demonstrating the effectiveness of our method and its safe applicability in a clinical context.

## 1. Introduction

Electroencephalography (EEG) is a widely used, non-invasive method for recording the electrophysiological activity of the brain and is a fundamental tool in the Neonatal Intensive Care Unit (NICU) for the real-time monitoring of the cerebral function of preterm and sick term neonates. Neonatal seizures are a commonly encountered neurologic condition in neonates that may have an adverse effect on neurodevelopment, with the risk of cognitive and behavioral disabilities or even epileptic outcomes in later life [1]. Cerebral function monitor generally detects only half of the neonatal seizures, which, therefore, need to be diagnosed, characterized, and quantified by means of the EEG [2,3,4,5,6,7]. The EEG has also been shown to be useful in quantifying neonatal stress [8] or estimating functional brain age [9,10] and predicting outcome [11,12,13].

However, EEG signals are often contaminated by artefacts of physiological origin that can hinder the true brain activity. Among those, artefacts originating from the cardiac activity may alter the results of the analysis of EEG signals, because their frequency range (i.e., 0.3–6 Hz) overlaps with the brain activity and their amplitude is often comparable to that of brain signals. In the case of neonatal EEG, these problems are even more severe because the frequency content of brain activity in newborns is typically below 5 Hz [14] and the amplitude of the EEG signals is very small. Moreover, the quality of the neonatal EEG is affected by the lower thickness and different structural compositions of the scalp and the skull, which produce inhomogeneities in the skull conductivity and, consequently, distortions in the EEG signals recorded at the scalp. The two fontanelles along the skull midline and the four open sutures in the temporo-parietal areas also contribute to major inhomogeneities in the electrical field at the neonatal head surface. The anterior fontanelle, which is close to the standard positions of the Cz and Fz electrodes, is generally the main cause of cardiac-related interference in neonatal EEG signals, including interference from the pulsatile activity of the underlying arterial vessels. For these reasons, the algorithms developed for the removal of cardiac artefacts in the adult EEG [15,16,17,18,19,20,21,22,23,24,25,26,27,28,29,30,31] cannot straightforwardly be applied to neonatal EEG recordings.

A reliable clinical analysis of the neonatal EEG, especially when performed with the aid of automated analytical methods, requires artefact-free EEG signals to be available [6,8,9,32,33]. Typically, neonatologists identify the EEG segments affected by artefacts of different types (such as muscular artefact, cardiac interference, eye movements and eyeblinks) by visual inspection and disregard them in the subsequent clinical evaluations, at the risk of losing important information on the neonatal brain function. Moreover, it should be noted that visual inspection is a time-consuming procedure—especially for the long neonatal EEG recordings that can last for several hours—requires specific expertise and is prone to subjective evaluations.

Therefore, it is recommended that automated methods for the detection and removal of artefacts from neonatal EEG recordings are developed and applied before any clinical analysis of the EEG. However, given the inherent difficulties in removing artefacts from the neonatal EEG, scientists have generally preferred to develop methods for the automated detection of segments affected by artefacts of several origins that are ignored in the subsequent clinical analysis of the EEG. Stevenson, Kauppila and Webb and their colleagues developed methods for the detection of artefactual segments in the neonatal EEG that were based on various types of EEG classification, without proposing a solution to the removal of the artefactual segments from the EEG [34,35,36].

Other authors attempted to develop automated methods able to not only detect but also remove cardiac interferences from the neonatal EEG. Their methods can be grouped in two classes: (1) methods based on filtering that exploit the simultaneously recorded electrocardiographic (ECG) signals; (2) methods based on Blind Source Separation (BSS) algorithms. The methods in the first class employ time or frequency features extracted from the ECG signals to detect the cardiac-related artefacts affecting the EEG recordings; after detection, these artefacts are eliminated through a weighted subtraction, usually by means of adaptive filtering [37,38] or filtering and Maching pursuit decomposition [33]. However, the main limitation of these methods is the non-optimal subtraction of the weighted cardiac interference due to the large amplitude variability in the real ECG signals. Consequently, these methods do not reach, in neonatal EEG, the same performances that are achieved in adult EEG.

The methods in the second class are based on BSS approaches, typically Independent Component Analysis (ICA) algorithms [38,39,40,41], which are widely applied in several adult studies [20,26,42,43,44,45,46,47,48,49,50]. However, the aforementioned inhomogeneities of the neonatal EEG and the similar amplitude and frequency content of the true brain activity and cardiac interference may reduce the effectiveness of ICA algorithms in separating artefactual signal components from components containing brain signals. Moreover, the decomposition power of any ICA algorithm is generally decreased by the low number of electrodes typically used for neonatal EEG recordings (from 8 to 19 channels). In fact, artefacts characterized by a low amplitude and a high degree of correlation across time, such as the cardiac and pulse interferences, typically spread across several independent components (ICs) and mix with the true brain activity, leading to a non-optimal source signal decomposition.

To improve the separation of cardiac and pulse interferences from the neonatal EEG, some researchers investigated the effectiveness of the most common ICA algorithms when artificial signals simulating the electrical cardiac activity and the pulsatile interference were added to the EEG signals [39,41,51]. Of all the tested algorithms (Robust ICA; fast fixed-point ICA, FpICA; AMUSE—Algorithm for Multiple Unknown Signals Extraction; SOBI—Second Order Blind Separation; JADE—Joint Approximation Diagonalization of Eigen-matrices; CCA—Canonical correlation analysis) SOBI had the best separation performance, reaching a correlation of 0.999 and 0.397 between the artefactual ICs and the spike signal simulating the electrical cardiac interference and the sinusoidal signal simulating the pulsatile interference, respectively. However, these authors focused on the separation performance of the ICA algorithms and did not assess the quality of the residual neonatal EEG signals.

De Vos et al. [13] investigated the performance of two specific ICA algorithms, RobustICA and SOBI, used for detecting the electrical cardiac and pulsatile interferences, respectively. The separation performance was considered good if the correlation between the artefactual ICs and the electrical cardiac and pulsatile interferences was greater than 0.6 and 0.4, respectively. The goodness of the proposed method was only assessed by verifying that the performance of a method for the automatic detection of seizures on the neonatal EEG improved after removal of the artefactual ICs, without verifying that the neonatal brain activity was preserved in the reconstructed EEG signals.

Based on these findings, when cardiac-related artefacts need to be removed, the use of the ICA algorithm with the best performance for neonatal EEG decomposition is not sufficient, and it must be supported by a procedure able to improve the separation of both the electrical cardiac and pulsatile interferences, hence reducing the number of mixed ICs that would affect the correct reconstruction of true brain signals. It is also worth mentioning that, when a BSS decomposition is applied to neonatal EEG recordings, the separated ICs are then classified as artefactual or not only by visual inspection. However, this is a time-consuming process that is also highly dependent on the experience of the operator. In a clinical scenario, the waste of time and any form of subjectivity in data interpretation should be minimized. Therefore, an automated procedure for the IC classification would be preferable.

We recently developed a method to automatically detect and remove cardiac-related artefacts from the adult EEG (Automatic Removal of Cardiac Interference (ARCI), [26,52]). This method employs ICA data decomposition and a set of defined data features, optimized to identify cardiac artefacts. Herein, we propose an extension of this method for the automated detection and removal of artefacts of cardiac origin (both electrical and pulsatile) from neonatal EEG recordings. Our method employs the ICA algorithm identified by other authors as optimal for neonatal applications (SOBI) and information on the time-domain features of both the electrophysiological activity of the heart and the pulsatile activity of blood vessels to optimize the separation of the related artefacts from true brain signals. Differently from similar methods using artificial cardiac and pulse signals [39,41], in our method, the time-domain features of the artefactual signals are extracted from the real ECG and simultaneously recorded with the EEG. The separated ICs are then automatically classified with a procedure based on our previous classification method (ARCI), optimized for neonatal applications. The goodness of the separation of the artefactual ICs from ICs containing true brain signals and the IC classification performance were evaluated statistically and by means of several quantities defined in the frequency domain. Finally, we also assessed the quality of the artefact-free neonatal EEG signals reconstructed from the retained non-artefactual ICs.

The main contributions of our method to the pre-processing of the neonatal EEG can be summarized as follows:

1. This is the first method that is able to automatically detect and remove both electrical cardiac and pulsatile interference while preserving the neonatal brain activity.
2. It can save time and reduce mistakes related to the expertise of the operator, who detects artefactual segments by visual inspection.
3. It employs ICA, which is a familiar tool for clinicians pre-processing EEG recordings.
4. It uses information derived from the real ECG—typically recorded simultaneously with the EEG in the NICU—to improve the separation performance of the ICA algorithm.
5. Its performance has been statistically assessed in terms of accuracy, sensitivity and false omission rate and in the quality of the reconstructed artefact-free EEG signals.
6. It can be used in conjunction with other methods dedicated to the correction of other types of artefacts and prior to analytical methods developed for diagnostic purposes (such as for the detection and classification of seizures).

## 2. Materials and Methods

### 2.1. EEG Recordings

The EEG dataset, obtained from a freely available online repository (Zenodo, https://zenodo.org/record/2547147#.YUr9by8QNUM (accessed on 5 June 2018) contains multi-channel EEG recordings from 79 human neonates, admitted to the NICU at the Helsinki University Hospital. The median recording duration was 74 min (Inter Quartile Range: 64 to 96 min). EEG signals were recorded with a NicOne EEG amplifier (Natus Medical Incorporated, Pleasanton, CA, USA) equipped with an EEG cap (Waveguard, ANT-Neuro, Hengelo, The Netherlands) mounting 19 sintered Ag/AgCl embedded electrodes that are arranged following the international 10–20 standard, with the recording reference at the midline position. The ECG was also recorded simultaneously with the EEG. All physiological signals were acquired with a sampling rate of 256 Hz.

From the complete EEG dataset of 79 neonatal recordings, 3 out of the 22 seizure-free multichannel EEG recordings were contaminated by both electrical cardiac and pulsatile interference (subject3, subject10, subject72). These seizure-free EEG recordings were selected to implement and test our method. To increase the statistical significance of our results, 20 neonatal EEG segments of 5 min duration were selected (6 segments from subject3; 8 segments from subject10; 6 segments from subject72).

To further test the efficacy and reliability of our proposed method, we applied it to multi-channel neonatal EEG recordings containing seizures, which were available from the same online repository. The seizures were annotated by three expert neonatologists. Out of the 57 EEG recordings with seizures, only 15 EEG recordings were contaminated by cardiac and pulse interference, as verified by an expert investigator by visually inspecting the time course and the power spectrum of each EEG channel. From these 15 multi-channel EEG recordings, 20 EEG segments of 5 min duration (10 segments with seizures and 10 without seizures) were selected to test our proposed method. The selected EEG segments with seizures were characterized by consistent seizure annotations of the three expert neonatologists.

### 2.2. EEG Data Analysis

**Figure 1:**
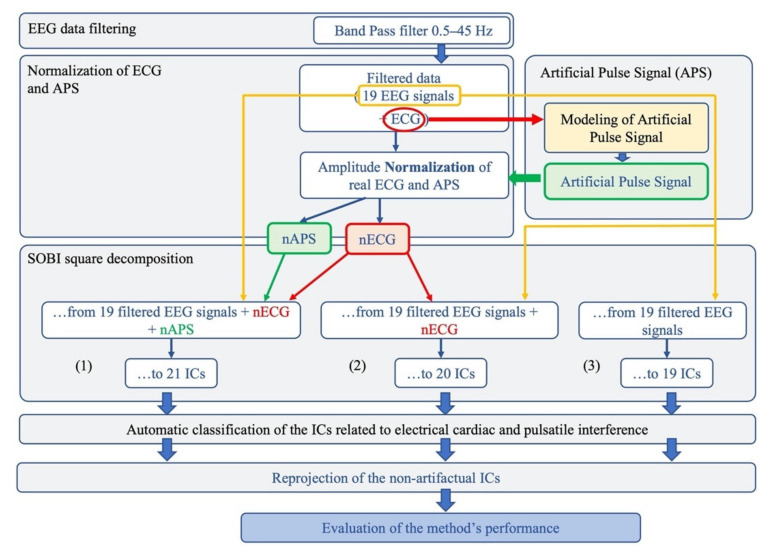
Flowchart of the complete data processing for the automated classification of cardiac-related interference ICs. The complete data-processing pipeline includes the sequential stages of data-processing and their respective modular processing steps, input and output.

The proposed method aims to identify and remove both electrical cardiac and pulsatile interference from neonatal EEG data. The basic idea was that, by adding both artefactual signals to the EEG recordings, the decomposition power of the BSS algorithm would increase, leading to a better separation of the artefactual components. The analysis pipeline is shown in Figure 1 and briefly described below.

First, the EEG recordings were band-pass filtered between 0.5 and 45 Hz by means of an FIR filter implemented in the EEGLAB toolbox [53,54]. By doing this, we retained the information on neonatal brain activity—whose frequency content is generally below 5 Hz—while removing high-frequency activity that is of no interest in neonatal studies [14]. Then, the ECG signal was used to build an Artificial Pulse Signal (APS) to be used as input to the BSS algorithm, together with the ECG and EEG signals. Due to the different amplitudes of the EEG and ECG signals (µV vs. mV), the cardiac and pulse signal were normalized to the average amplitude of the EEG signals. To test whether the separation of the artefactual components improved with the inclusion of the normalized ECG and APS signals as input signals to the BSS algorithm, three groups of signals were separately considered for validating our method, including the 19 filtered neonatal seizure-free EEG signals, together with the normalized ECG and APS signals, or the EEG and the normalized ECG only, or just the EEG signals (Figure 1).

For each signal group, the separated ICs were classified as artefactual (cardiac/pulse) or non-artefactual. An automated classification procedure was implemented to recognize the features of pulse (labeled as Pulse Cardiac Component, PCC) or heart (labeled as Electrical Cardiac Component, ECC) activity. The automated procedure was compared with a classification based on the visual inspection of the topography, time course and Power Spectral density of each IC, performed independently by two expert investigators. Finally, for each neonatal EEG dataset, the ICs classified as artefactual were disregarded and the artefact-free EEG signals were reconstructed by re-projecting all the retained non-artefactual ICs onto the sensor space.

This procedure was repeated for the EEG segments extracted from the neonatal EEG recordings containing seizures, with the only exception being that, in this case, only one group of input signals to the BSS algorithm was considered, including the 19 filtered neonatal EEG signals together with the normalized ECG and APS signals (group G1s).

All EEG data preprocessing and decomposition was performed using EEGLAB toolbox (v. 13.6.5b, Swartz Center for Computational Neuroscience, La Jolla, CA, USA [54]) operating in the MATLAB environment (release MatlabR2018b).

#### 2.2.1. Artificial Pulse Signal

**Figure 2:**
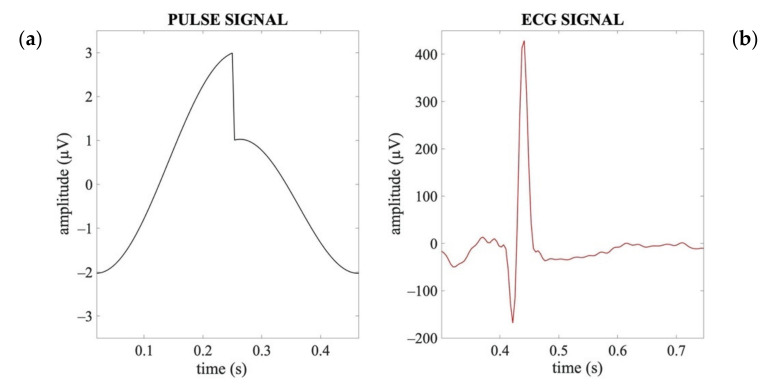
Exemplary waveforms of an artificial pulse signal (**a**) and an ECG signal (**b**) in a time interval corresponding to one heartbeat period.

Although the ECG and pulse signals share common spectral properties (i.e., heart rate), they differ in their waveform. The pulse signal has a waveform characterized by a high amplitude and slow temporal progression like a saw-tooth wave (Figure 2a), whereas the ECG is composed of a sequence of waves, of which the one corresponding to the ventricular depolarization is very rapid and dominant in terms of amplitude (R peak in the ECG signal, Figure 2b).

Moreover, according to the electrophysiology of the heart and the blood pressure dynamics, the cardiac signal associated with the electrical activity of the heart typically precedes the signal associated with the pulsatile activity in the main blood vessels by about 200–300 ms [55,56,57].

**Figure 3:**
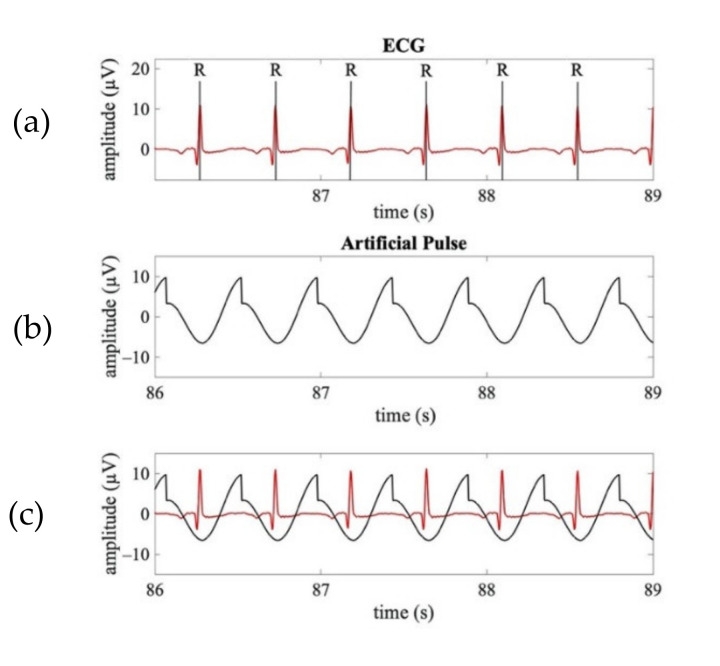
Examples of a real ECG signal synchronously acquired with the EEG signals and the corresponding APS. (**a**) Time course of a real ECG signal (3 s). (**b**) Time course of the APS corresponding to ECG shown in (**a**). (**c**) Time course of the superimposed real ECG (red line) and APS (black line).

The template waveform of a typical APS (shown in Figure 2a) was obtained by a combination of a saw-tooth wave and a cosine wave. Given the time-delay between the pulsatile and electrical cardiac activities, the synthetized APS for each neonate was obtained as a sequence of templates with the maximum amplitude aligned at 250 ms after the R peaks identified on the ECG signal (Figure 3).

#### 2.2.2. Normalization of the ECG and APS signals

Each neonatal EEG recording was composed of 19 EEG signals and 1 ECG signal. To obtain a good signal separation, it is necessary for the input signals to the BBS algorithm to have an average amplitude of the same order of magnitude. Therefore, given that the amplitude of the ECG is typically three orders of magnitude larger than the amplitude of the EEG signals (mV vs. µV), the ECG signal was normalized with respect to the mean peak-to-peak amplitude of the 19 filtered EEG signals (MAmpEEG) according to the following equation:

(1) $$\mathrm{nECG}\left(k\right)=\frac{\mathrm{ECG}\left(k\right)\times \mathrm{MAmpEEG}}{\mathrm{AmpECG}\left(k\right)}$$

where nECG is the normalized ECG, k indicates a time point, and AmpECG is the peak-to-peak amplitude of ECG signal.

The same procedure was followed for the normalization of APS, where the template was scaled to the average amplitude of the EEG signals to obtain normalized APS signals (nAPS).

#### 2.2.3. BBS Decomposition

Signal decomposition for the separation of the artefactual components (electrical cardiac and pulsatile) was performed by applying SOBI because it was demonstrated to have the best performance in the separation of cardiac interference from neonatal EEG signals [13,39,41,51,58].

To test whether the separating power of SOBI increased by adding the normalized ECG and APS signals to the input EEG signals, for each neonatal EEG segment of the seizure-free EEG recordings, we considered three different groups of signals:

1. Group 1 (G1): 21 signals, including the 19 filtered neonatal EEG signals and the normalized APS and ECG signals;
2. Group 2 (G2): 20 signals, including the 19 filtered neonatal EEG signals and the normalized ECG signal;
3. Group 3 (G3): 19 signals, including only the 19 filtered neonatal EEG signals.

SOBI is based on a joint diagonalization of correlation matrices. Therefore, a square decomposition was applied, that, depending on the group of signals considered, resulted in 21 ICs (G1), 20 ICs (G2) and 19 ICs (G3).

The neonatal EEG segments selected from the EEG recordings containing seizures composed a fourth group (G1s) of 21 signals, including the 19 filtered neonatal EEG signals and the normalized APS and ECG signals. In this case, the decomposition of the input signals resulted in 21 ICs.

#### 2.2.4. Automated Classification of the ICs Containing Cardiac Electrical and Pulsatile Interferences

The automated classification of the artefactual ICs containing cardiac electrical and pulsatile interferences was based on the comparison of the features in the time and frequency domain between the normalized cardiac/pulse signals (nECG/nAPS) and the separated ICs. The Power Spectral Density (PSD) of each component was calculated from 0.5 to 45 Hz with a standard Welch procedure (Hamming windowing, frequency resolution of 0.01 Hz) and used in the automatic classification procedure, as described below.

To verify whether an IC contained cardiac electrical or pulsatile interferences, we first assessed the correlation between the PSD of the IC and the PSD of the nECG or nAPS signal and the correlation between the time course of the IC and the time course of the nECG or nAPS signal. Correlation was calculated by means of the Pearson correlation coefficient (*r*). Given that the neonatal EEG signals can be very noisy, we imposed that at least one of these correlation coefficients must be very high (>0.9), whereas the other is considered sufficiently good if it is greater than 0.75. If one of these two sets of conditions (i.e., either *r*_PSD_ > 0.9 and *r*_time-course_ > 0.75 or *r*_PSD_ > 0.75 and *r*_time-course_ > 0.75) was met, then the IC was classified as an electrical cardiac component (ECC) or a pulsatile cardiac component (PCC), respectively, for the cardiac electrical or pulsatile interference.

If none of these two sets of conditions were met, we considered that, for an IC to be artefactual (either an ECC or a PCC), the condition that *r*_PSD_ must be greater than 0.9 should be satisfied along with another condition, specific to each type of artefact:

1. For the cardiac electrical interference: the time delay between the peaks of the IC and the peaks of the nECG signal must be equal to 0;
2. For the pulsatile interference: the time delay between the peaks in the IC and the peaks in the nECG signal must be included between 150 and 350 ms [55,56,57].

If the IC did not meet any of the sets of conditions described above, it was classified as a non-cardiac component (NCC).

**Figure 4:**
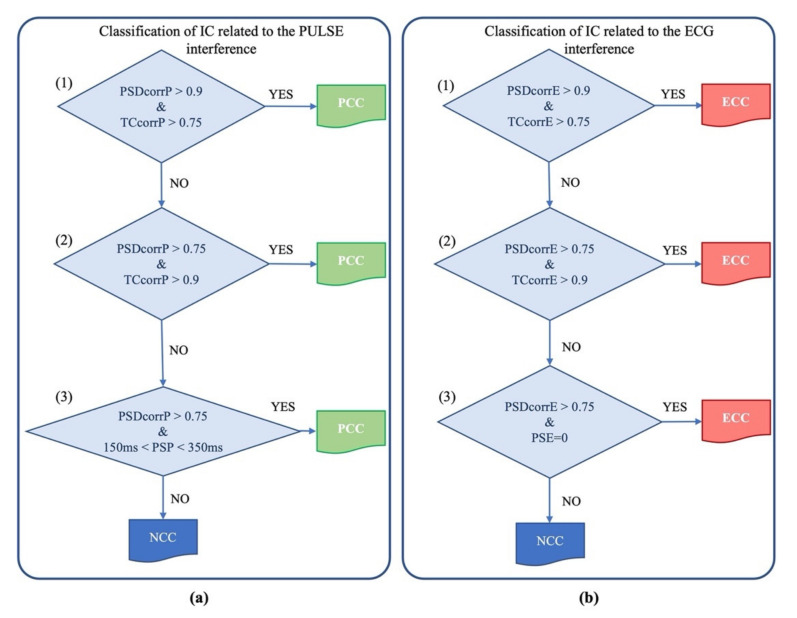
Flux diagram of the automated process for the classification of the artefactual component as PCC (**a**) and ECC (**b**) through a cascade of three criteria. Acronyms: PSDcorrP: correlation value between the component PSD and the APS PSD; TCcorrP: correlation value between the time course of the component and the time course of APS; PSP: Phase Shift value between the component and APS; PSDcorrE: correlation value between the component PSD and the ECG PSD; TCcorrE: correlation value between the time course of the component and the time course of real ECG; PSE: Phase Shift value between the component and the ECG.

The procedure for the automated classification of the artefactual ICs is graphically described in Figure 4.

### 2.3. Evaluation of the Method’s Effectiveness

#### 2.3.1. Evaluation of the Influence of Using the nECG and nAPS Signals in the SOBI Decomposition

As described in Section 2.2.3., for each neonatal EEG segment of the seizure-free EEG recordings, the decomposition procedure using the SOBI algorithm was performed on three different groups of signals (G1, G2 and G3). To assess whether the decomposition power of SOBI was improved by adding the nECG and the nAPS to the input EEG signals, we calculated three quantities, as described below.

The major issue encountered when applying BSS methods to neonatal EEG datasets for separating cardiac-related artefacts is that these artefacts are generally not included in few ICs, but are spread over all separated ICs, mixing with true brain activity. To verify the effectiveness of our approach in reducing the number of ICs containing cardiac-related interferences (i.e., both cardiac electrical and pulsatile artefacts), we calculated the percentage of ICs that had a non-negligible power at the frequency of the neonatal heart rate for the three signal groups G1, G2 and G3.

For each EEG recording, we first calculated the heart rate frequency from the simultaneously recorded ECG, which, in neonates, is generally around 2.4 Hz [59,60]. Then, from the PSD of each IC, we first calculated the power at the specific neonatal heart rate frequency and then normalized it to the total power at that frequency across all separated ICs. This normalization was necessary to compare the results obtained for the three signal groups G1, G2 and G3. This quantity, called Artefactual Percentage Index of Components (APICs), was calculated according to Equation (2):

(2) $${\mathrm{APICs}}_{j}=\frac{{\mathrm{cPSDhr}}_{j}}{\sum_{i=1}^{N}{\mathrm{cPSDhr}}_{i}}\times 100$$

where cPSDhr is the power at the neonatal heart rate frequency, and N is the total number of ICs in the signal group (i.e., 21, 20 and 19 for G1, G2 and G3, respectively).

To see whether our method permitted a reduction in the number of ICs containing cardiac-related interferences, for each neonatal EEG segment and for each signal group G1, G2 and G3, we then calculated the percentage of ICs with APICs_j_ > 1%. This quantity, called Contaminated Components (CC), was calculated according to Equation (3):

(3) $$\mathrm{CC}=\frac{K}{N}$$

where K is the number of ICs with APICs_j_ > 1% and N is the total number of separated ICs.

Afterwards, to assess whether a major part of the power related to cardiac electrical and pulsatile interferences is contained in the ICs classified as ECC or PCC, we calculated, for each EEG recording and signal group, the total amount of the signal power at the heart rate frequency in the ICs, classified as ECC or PCC. This quantity, called Artefactual Percentage Index (API), is given by:

(4) $$\mathrm{API}=\overset{N_{A}}{\underset{j=1}{\sum }}\left({\mathrm{APICs}}_{j}\right)$$

where APICs_j_ is defined in Equation (2) and N_A_ is the number of artefactual ICs.

A high value of API indicates that the cardiac-related power is mainly contained in the ICs classified as ECC or PCC.

The quantities CC and API were also calculated for the EEG segments separated from the neonatal EEG recordings containing seizures (group G1s, 21 ICs).

Finally, for each EEG recording and signal group (G1, G2, G3 and G1s), we calculated the number of ICs that were automatically classified as ECC or PCC, respectively.

#### 2.3.2. Evaluation of the Quality of the Reconstructed EEG Signals

The effectiveness of the proposed method in removing cardiac-related interferences by means of few artefactual ICs would influence the possibility of better preserving the true brain activity in the EEG signals reconstructed from the retained ICs. The quality of the reconstructed EEG signals was assessed by calculating three parameters, as described below.

First, for each neonatal EEG segment and decomposition level (i.e., decomposition to 21, 20 and 19 ICs for G1 and G1s, G2 and G3, respectively), the total power of the EEG signals was calculated before and after removing the artefactual ICs (both ECC and PCC). The percentage average variation in the total power of the EEG signals (Signal Power Variation, SPV) was calculated as:

(5) $$\mathrm{SPV}=\frac{\sum_{i=1}^{19}{\left(\frac{{\mathrm{PowerEEG}}_{b}-{\mathrm{PowerEEG}}_{a}}{{\mathrm{PowerEEG}}_{b}}\times 100\right)}_{i}}{19}$$

where PowerEEG_b_ and PowerEEG_a_ are the signal powers before and after artefactual ICs removal and *i* denotes the *i*th EEG signal. Please note that SPV was normalized to 19 channels for the signal groups G1, G2 and G3 because it was calculated for the EEG signals only. For the signal group G1s, only the EEG signals affected by cardiac and/or pulse interference were considered, because not all channels in this group of EEG recordings were affected by cardiac-related artefacts.

Second, we calculated the total EEG signal power at the R peaks, as identified on the ECG. In particular, for each neonatal EEG segment and signal group (i.e., G1, G1s, G2 and G3), we calculated the average over all EEG channels of the square amplitudes of the EEG signals at the R peaks, normalized by the number of R peaks. A quantity, called variation of R Peak Power (varRPP) and calculated according to Equation (6), provides an estimate of the percentage reduction in the EEG signal power at the R peaks after artefact removal:

(6) $$\mathrm{varRPP}=\frac{\sum_{i=1}^{19}{\left(\frac{\sum_{j=1}^{N_{R}}\frac{{\mathrm{RPP}}_{b}-{\mathrm{RPP}}_{a}}{{\mathrm{RPP}}_{b}}\times 100}{N_{R}}\right)}_{i}}{19}$$

where RPP_b_ and RPP_a_ are the EEG signal powers at the R peaks before and after artefact removal, respectively; *i* denotes the *i*th EEG signal; *j* denotes the *j*th R peak; N_R_ is the number of R peaks. Please note that varPP is normalized to 19 for all signal groups (G1, G2 and G3) because varRPP is calculated for the EEG signals only.

Finally, for each neonatal EEG segment of the seizure-free EEG recordings and signal group, we calculated the variation in the EEG signal power at the specific heart rate frequency. In fact, the removal of artefactual ICs (both ECC and PCC) should lead to a reduction in the PSD at the heart rate frequency in the reconstructed EEG signals. Therefore, we calculated the percent variation in the difference of the average power at heart rate frequency across all EEG channels before and after removal of the artefactual ICs. The quantity varPSDhr (i.e., the variation in the PSD at the heart rate frequency) was calculated as:

(7) $$\mathrm{varPSDhr}=\frac{\sum_{i=1}^{19}{\left(\frac{{\mathrm{PSDhr}}_{b}-{\mathrm{PSDhr}}_{a}}{{\mathrm{PSDhr}}_{b}}\times 100\right)}_{i}}{19}$$

where PSDhr_b_ and PSDhr_a_ are the PSD amplitude at the heart rate frequency before and after artefact removal, respectively, and *i* denotes the *i*th EEG signal. Please note that varPSDhr is normalized to 19 channels for the signal groups G1, G2 and G3 because it is calculated for the EEG signals only. For the signal group G1s, only the EEG signals affected by cardiac and/or pulse interference were considered, because not all channels in this group of EEG recordings were affected by cardiac-related artefacts.

### 2.4. Statistical Analysis

#### 2.4.1. Assessment of the Effectiveness of the Proposed Decomposition Method for Separating Cardiac-Related ICs with the Three Signal Groups Composed with Seizure-Free EEG Segments (G1, G2 and G3) and with the Signal Group of EEG Segments with Seizures (G1s)

The impact of using the nECG and nAPS signals, together with the EEG signals, on the SOBI decomposition effectiveness was evaluated. To this aim, we compared the six quantities defined in Section 2.3.1 and Section 2.3.2 (i.e., CC, API, number of artefactual Ics—both ECC and PCC, SPV, varRPP, varPSDhr) across the three signal groups (G1, G2 and G3). As each of these quantities had a non-normal distribution, a Friedman test was performed to verify the differences across signal groups. When the test was significant, a series of two tailed Wilcoxon’s signed rank tests were performed as post-hoc comparisons. For each comparison, the r effect size proposed by Cohen [61,62,63] was calculated. Effect size values of 0.10, 0.30, and 0.50 indicate small, medium and large effects, respectively [62]. The significance level was set at 0.05. Statistical analysis was performed using the Statistical Package for Social Sciences software (SPSS v. 25, IBM, Armonk, New York, NY, USA). The effectiveness of using the nECG and nAPS signals together with the EEG signals was also assessed in the group of EEG segments with seizures (G1s). To this aim, we compared the distribution of CC, API, SPV, varRPP and varPSDhr obtained for the group of EEG signals in G1s with those obtained for G1, given that, in both cases, the input signals were separated in 21 ICs.

#### 2.4.2. Validation of the Automated Classification of the ICs: Comparison with the Expert Classification of the ICs

To assess the overall classification performance of our method, for each signal group (G1, G2, G3 and G1s), we compared the automated IC classifications with the IC classifications made by two independent experienced investigators. For both classifications, ECCs and PCCs were merged to form a unique set of cardiac components (Cardiac Related Components, CRCs), as was previously carried out in [26]. To compare the two methods, we calculated:

(1) True Positives (TP): the number of artefactual ICs correctly classified as CRCs by the algorithm;
(2) True Negative (TN): the number of non-artefactual ICs correctly classified as NCCs by the algorithm;
(3) False Negative (FN): the number of artefactual ICs wrongly classified as non-artefactual (i.e., as NCCs) by the algorithm;
(4) False Positive (FP): the number of non-artefactual ICs wrongly classified as artefactual (i.e., as CRCs) by the algorithm.

According to the validated procedures [26,42,64], the classification performance was evaluated by means of three statistical measures: accuracy, False Omission Rate (FOR) and sensitivity, defined according to the following equations:

(8) $$\mathrm{Accuracy}=\frac{\sum \left(\mathrm{TP}+\mathrm{TN}\right)}{\sum \left(\mathrm{TP}+\mathrm{TN}+\mathrm{FP}+\mathrm{FN}\right)}$$

(9) $$\mathrm{FOR}=\frac{\sum \mathrm{FN}}{\sum \left(\mathrm{TN}+\mathrm{FN}\right)}$$

(10) $$\mathrm{Sensitivity}=\frac{\left(\frac{\sum \mathrm{TP}}{\sum \left(\mathrm{TP}+\mathrm{FN}\right)}\right)-\left(\frac{\sum \mathrm{FP}}{\sum \left(\mathrm{FP}+\mathrm{TN}\right)}\right)}{1-\left(\frac{\sum \mathrm{FP}}{\sum \left(\mathrm{FP}+\mathrm{TN}\right)}\right)}$$

## 3. Results

### 3.1. Testing of the Proposed Method on the Seizure-Free EEG Recordings

**Figure 5:**
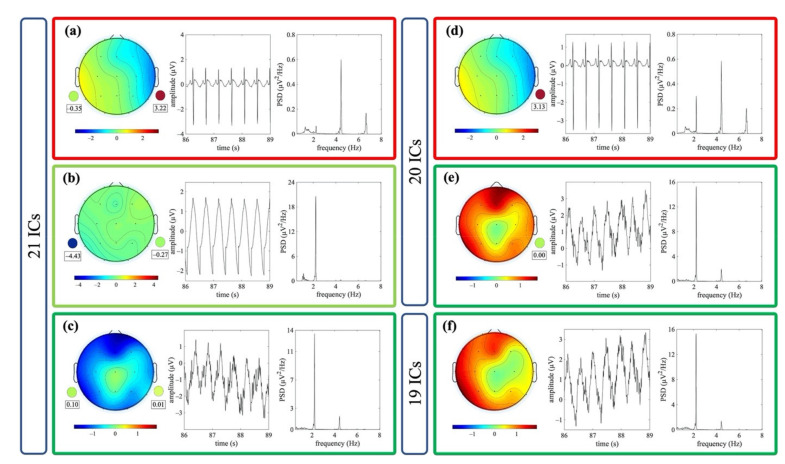
Exemplary components classified as artefactual in the three signal groups (i.e., decomposition to 21, 20 and 19 ICs for G1, G2 and G3, respectively). Examples refer to the same EEG segment. Scalp topography, 3 s of time course and PSD of one IC classified ECC (**a**) and of two ICs classified as PCC (**b**,**c**) are shown for the decomposition to 21 ICs. The weights of the mixing matrix corresponding to the nECG and nAPS signals are represented by circles (pulse activity on the left, heart activity on the right). Scalp topography, 3 s of time course and PSD of one IC classified as ECC (**d**) and one IC classified as PCC (**e**) are shown for the decomposition to 20 ICs. The weights of the mixing matrix corresponding to the nECG is represented by a circle. In (**f**), the scalp topography, 3 s of the time course and PSD of one IC classified as PCC is shown for the decomposition to 19 ICs.

Figure 5 shows an example of ICs classified as ECC or PCC for one dataset. The outcome of the decomposition using the different signal groups G1, G2 and G3 is also shown.

**Figure 6:**
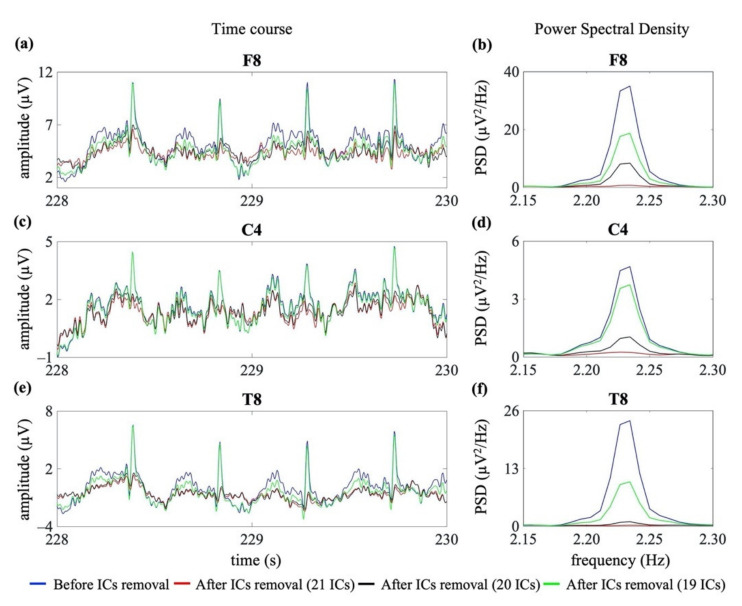
Examples of cardiac artefact removal in three EEG channels (frontal: F8, central: C4 and temporal: T8). Panels (**a**,**c**,**e**) show 2 s of the time course of three exemplary EEG signals from one neonatal EEG segment. Panels (**b**,**d**,**f**) show the PSD around the specific heart rate frequency (i.e., 2.23 Hz). The blue lines indicate the PSD and time course before the removal of cardiac-related interference. The red, black and green lines indicate the PSD and time course after removal of the cardiac-related interference using the three signal groups, i.e., decomposing to 21 ICs, 20 ICs and 19 ICs.

Figure 6 shows an example of the removal of the cardiac interference (both cardiac electrical and pulsatile) from three EEG channels of the same EEG segment, shown in Figure 5 (right frontal: F8; right central: C4; right temporal: T8), using the three signal groups G1, G2 and G3 (hence decomposing to 21 ICs, 20 ICs and 19 ICs, respectively).

#### 3.1.1. Evaluation of the Effectiveness of Using the nECG and nAPS Signals in the SOBI Decomposition

**Figure 7:**
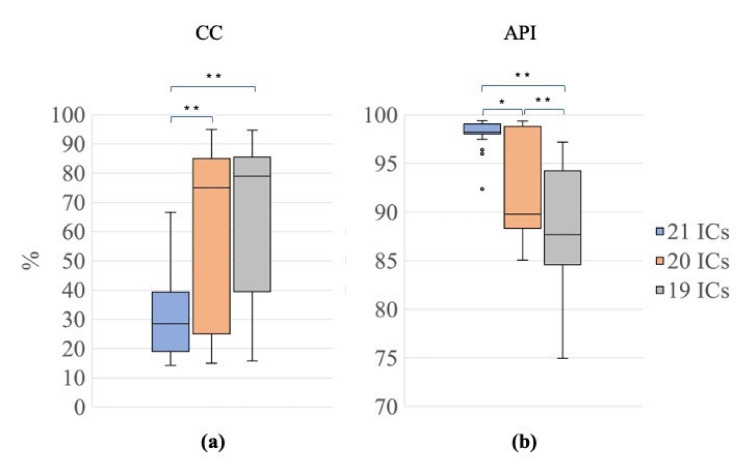
Distribution of CC and API for the different decompositions. Box plots of the percentage of contaminated ICs (CC, (**a**)) and of the Artefactual Percentage Index (API, (**b**)) for the three signal groups (G1, 21 ICs: blue, G2, 20 ICs: orange; G3, 19 ICs: grey). The horizontal black lines, the boxes, the vertical bars, and the dots indicate the median, the 25–75 percentile, the 5–95 percentile and the outliers, respectively. Single and double asterisks indicate significant differences between signal groups with *p* < 0.05 and *p* < 0.001, respectively, as assessed by the Wilcoxon test.

Figure 7 shows the results obtained for CC and API to assess whether the use of the nECG and nAPS as input signals, together with the EEG signals, improved the decomposition power of the SOBI algorithm. The Friedman tests revealed a significant difference among the three signal groups (G1, 21 ICs; G2, 20 ICs; G3, 19 ICs) for both CC and API (Friedman test: χ^2^ = 21.70, *p* < 0.001 for CC; χ^2^ = 32.70, *p* < 0.001 for API). Post hoc Wilcoxon tests revealed that the CC values were significantly lower for G1 than for G2 and G3 (Wilcoxon test: 21 ICs vs. 20 ICs: Z = 3.66, *p* <0.001, r = 0.58; 21 ICs vs. 19 ICs: Z = 3.77, *p* < 0.001, r = 0.60; Figure 7a), indicating that the use of nECG and nAPS systematically permitted a reduction in the number of ICs containing cardiac-related interferences. Furthermore, the API values were higher for G1 than for G2 and G3 (Wilcoxon test: 21 ICs vs. 20 ICs: Z= 3.47, *p* = 0.001, r = 0.55; 21 ICs vs. 19 ICs: Z= 3.92, *p* <0.001, r = 0.62; 20 ICs vs. 19 ICs: Z = 3.88, *p* <0.001, r = 0.61, Figure 7b), showing that the ICs classified as ECC or PCC after the decomposition using the nECG and nAPS signals contained the majority of the power related to the cardiac electrical and pulsatile interferences.

Table 1 summarizes the results of the Friedman test and the Wilcoxon tests. The number of ICs classified as ECC and PCC was significantly different among the three signal groups (21 channels for G1, 20 channels for G2 and 19 channels for G3) (Friedman test: *ECC:* χ^2^ = 14.70, *p* < 0.001; *PCC:* χ^2^ = 19.43, *p* < 0.001; see Table 1). The Wilcoxon tests showed that the number of ICs classified as ECC were significantly different (*p* < 0.001) for the comparisons between the classifications made with 21 ICs and 20 ICs and that made with 19 ICs. Regarding the number of ICs classified as PCC, the Wilcoxon tests showed that all pairwise comparisons were significantly different, at least with *p* < 0.05 (Table 1).

#### 3.1.2. Evaluation of the Quality of the Reconstructed EEG Signals

The Friedman tests performed on SPV, varRPP and varPSDhr, that evaluate the quality of the EEG signals reconstructed after the decomposition with the three signal groups (G1, 21 ICs; G2, 20 ICs; G3, 19 ICs), showed significant differences among all signal groups (Friedman test: SPV: χ^2^ = 6.30, *p* = 0.040; varRPP: χ^2^ = 9.10, *p* = 0.011; varPSDhr: χ^2^ = 32.50, *p* < 0.001).

**Figure 8:**
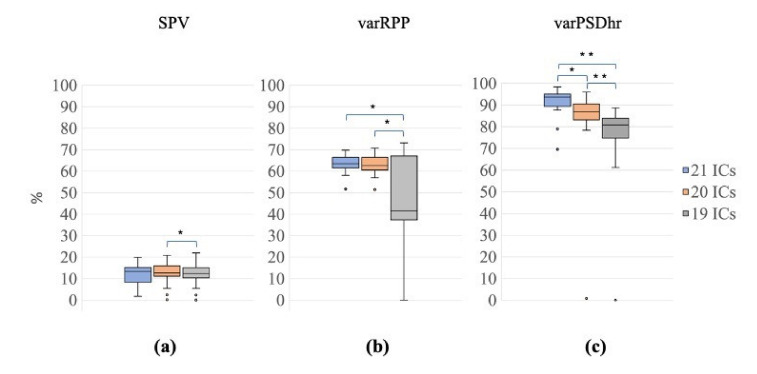
Distribution of SPV, varRPP and varPSDhr for the different decompositions. Box plots of percentage of SPV (**a**), varRPP (**b**) and varPSDhr (**c**) for the three signal groups (21 ICs: blue, 20 ICs: orange; 19 ICs: grey). The horizontal black lines, the boxes, the vertical bars, and the dots indicate the median, the 25–75 percentile, the 5–95 percentile and the outliers, respectively. Single and double asterisks indicate significant differences between the different signal groups with *p* < 0.05 and *p* < 0.001, respectively, as assessed by the Wilcoxon test.

The Wilcoxon tests for SPV revealed a significant difference only between G2 and G3 (i.e., between the decompositions with 20 ICs and 19 ICs) (Z = 2.80, *p* = 0.005, r = 0.44, Figure 8a).

Conversely, the Wilcoxon tests on varRPP showed significant differences between G3 and G1/G2 (G1 vs. G3: Z = 3.21, *p* = 0.001, r = 0.51; G2 vs. G3*:* Z = 3.14, *p* = 0.002, r = 0.50, Figure 8b). The difference between G1 and G2 approached significance (G1 vs. G2: Z = 1.94, *p* = 0.052, r = 0.31).

The Wilcoxon tests for varPSDhr also yielded significant differences among all signal groups (G1 vs. G2: Z = 3.21, *p* = 0.001, r = 0.51; G1 vs. G3: Z = 3.92, *p* < 0.001, r = 0.62; G2 vs. G3: Z = 3.92, *p* < 0.001, r = 0.62, Figure 8c).

#### 3.1.3. Validation of The Automated Classification of the ICs in Comparison with the Expert ICs Classification

The performance of our method in classifying the cardiac-related interferences was statistically assessed according to the decomposition level (21, 20, 19 ICs, i.e., G1, G2 and G3). The results are summarized in Table 2. The decomposition performed using the signal group G1 (i.e., using nECG and nAPS together with the EEG signals) showed a higher accuracy and sensitivity and a lower FOR than the other two groups (G2 and G3).

### 3.2. Validation of the Proposed Method on the EEG Recordings Containing Seizures

The method proposed for removing cardiac electrical and pulsatile interference from neonatal EEG recordings was further validated in a group of 20 EEG segments selected from 15 EEG recordings affected by seizures (group G1s, see Section 2.1 EEG recordings). For these EEG segments, we only considered the decomposition into 21 ICs of 21 input signals (i.e., the 19 EEG segments, the nECG and the nEPS).

**Figure 9:**
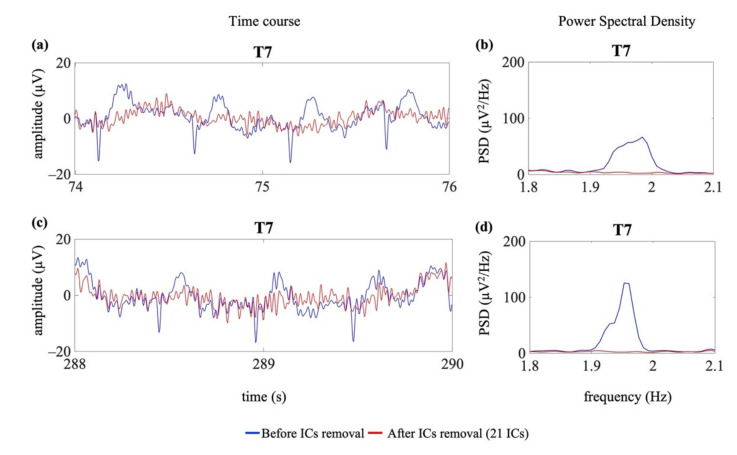
Examples of cardiac artefact removal in two EEG segments from one channel (T7) of an EEG recording containing seizures (G1s). Panel (**a**) shows 2 s of the time course of the EEG signal where no seizures were annotated, whereas panel (**c**) shows 2 s of the time course of the EEG signal during seizure. Panels (**b**,**d**) show the PSD around the specific heart rate frequency (i.e., 1.99 Hz (**b**) and 1.95 Hz (**d**)). The blue lines indicate the time course and the PSD before the removal of the cardiac-related interference. The red lines indicate the time course and the PSD after removal of the cardiac-related interference (decomposition into 21 ICs).

Figure 9 shows an example of the removal of the cardiac electrical and pulsatile interference from two EEG segments selected from EEG recordings affected by neonatal seizures (group G1s).

**Figure 10:**
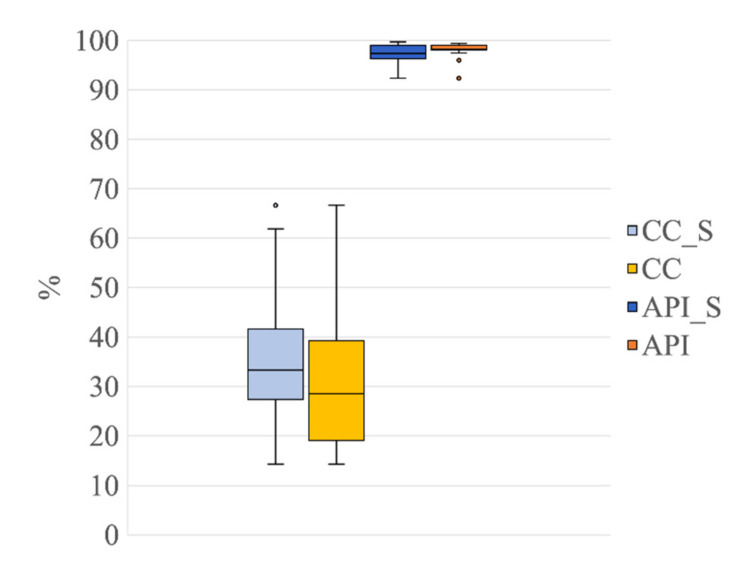
Distribution of CC and API for the group G1s in comparison to group G1 (both groups separated into 21 ICs). Box plots of the percentage of contaminated ICs (CC_S, in light blue, refers to G1s; CC, in light yellow, refers to G1) and of the Artefactual Percentage Index (API_S, in blue, refers to G1s and API, in yellow, refers to G1). The horizontal black lines, the boxes, the vertical bars, and the dots indicate the median, the 25–75 percentile, the 5–95 percentile, and the outliers, respectively.

Figure 10 shows the results obtained for CC and API for the group G1s in comparison with the results obtained for the group G1 (the latter already shown in Figure 7). Displaying the results of G1s and G1 together highlights any possible differences in the decomposition power of the SOBI algorithm when it is applied to EEG signals containing seizures in comparison to when it is applied to seizure-free EEG segments. The Wilcoxon tests, performed to establish whether there were any differences between G1s and G1 for CC and API, revealed no significant difference between the two groups for both metrics (CC: Z = 1.09, *p* = 0.28, r = 0.17; API: Z = 0.97, *p* = 0.33, r = 0.15; Figure 7a). This result indicates that the use of nECG and nAPS permitted a reduction in the number of ICs containing cardiac-related interferences in a similar way as the two groups of EEG signals, which means that the presence of seizures did not affect the effectiveness of the decomposition when 21 input signals were used.

**Figure 11:**
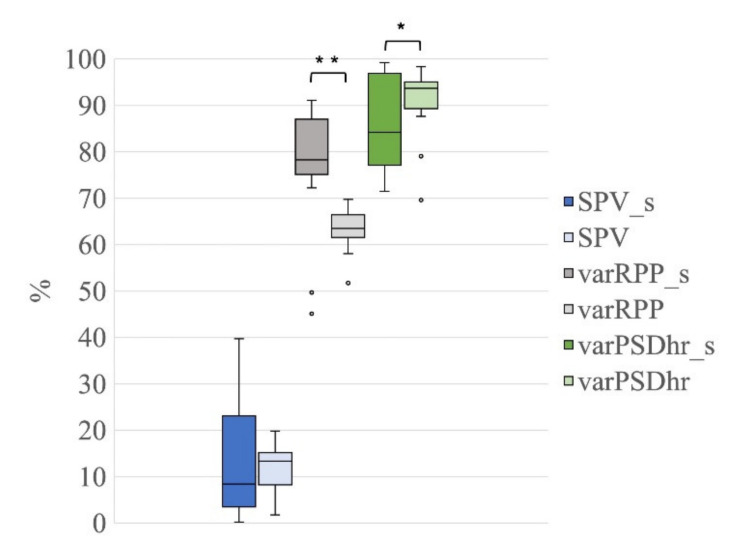
Distribution of SPV, varRPP and varPSDhr for the group G1s in comparison to group G1 (both groups separated into 21 ICs). Box plots of percentage of SPV (blue for G1s and light blue for G1), varRPP (dark grey for G1s and light grey for G1) and varPSDhr (dark green for G1s and light green for G1). The horizontal black lines, the boxes, the vertical bars, and the dots indicate the median, the 25–75 percentile, the 5–95 percentile and the outliers, respectively. Single and double asterisks indicate significant differences between the G1s and G1 with *p* < 0.05 and *p* < 0.001, respectively, as assessed by the Wilcoxon test.

When evaluating the quality of the reconstructed EEG signals, the Wilcoxon tests on SPV revealed no significant differences between G1s and G1 (Z = 0.22, *p* = 0.83, r = 0.04, Figure 11), meaning that the average percentage variation in the total power of the EEG signals is similar in the two groups and the presence of seizures does not affect this. Conversely, the Wilcoxon tests on varRPP and varPSDhr showed significant differences between G1s and G1 (varRPP*:* Z = 3.58, *p* = 0.000, r = 0.57; varPSDhr*:* Z = 1.98, *p* = 0.048, r = 0.31, Figure 11).

The significantly higher reduction in the signal power at the R peak frequency (higher varRPP) observed for G1s in comparison with G1 can be ascribed to the higher amplitude of the R peaks of the electrical cardiac interference in the EEG segments of G1s with respect to G1. Furthermore, given that the electrical cardiac and pulse interference have the same peak frequency (i.e., the R Peak frequency), the significant difference observed for the distribution of the varPSDhr values between G1s and G1 can be ascribed to the larger distribution of the varPSDhr values for G1s, most likely due to the higher number of EEG segments affected by both cardiac and pulsatile interference in G1s with respect to G1 (14 out 20 EEG segments for G1s vs. 9 out of 20 EEG segments for G1). Finally, it is worth observing that the distributions of SPV, varRPP and varPSDhr for G1s are more spread than those for G1, most likely because of the higher background noise affecting the EEG segments in G1s.

The classification performance of our method in G1s was also assessed in comparison with the expert ICs classification. The results are summarized in Table 3. The decomposition performed on the signal group G1s showed a higher accuracy and sensitivity and a lower FOR than in group G1, meaning that the separation performance of SOBI when the nECG and nAPS are given as input together with the EEG signals is also very good for the EEG signals affected by seizures.

## 4. Discussion

In this study, we optimized an ICA-based method for the automated detection and removal of cardiac-related interferences from neonatal EEG recordings. Cardiac interference is one of the most difficult artefacts to remove from neonatal EEG for several reasons: its amplitude, comparable with that of the EEG signals; the overlap of its main frequencies with the frequency content of neonatal brain activity; the contamination of many EEG channels because of the conductivity profiles of the neonatal skull and scalp and the presence of the fontanelles and open sutures; the low decomposition power of BSS methods with a small number of input channels.

The novelty of our method with respect to the approaches already proposed to remove cardiac interference from neonatal EEG signals resides in the exploitation of the time and frequency features of the real ECG signals, recorded simultaneously with the neonatal EEG when infants are hospitalized in the NICU. This approach, by adding the nECG and nAPS to the pool of neonatal EEG signals used as input to the SOBI algorithm, improved its decomposition performance and led to the separation of the electrical cardiac and pulsatile interferences in few ICs. Indeed, when using the nECG and nAPS signals together with the EEG signals, a very high percentage of power related to cardiac activity was contained in the few ICs classified as artefactual (API = 98.22% and 97.40% in G1 and G1s, respectively, Figure 7 and Figure 10). Please note that this result was independent of the type of EEG signal processed, i.e., whether it contained seizures or not. The percentage of cardiac and/or pulsatile signal power contained in the ICs classified as artefactual decreased substantially when only the nECG and the EEG signals or only the EEG signals were used (G2: API = 89.78%, G3: API = 87.69%). This result is associated with the fact that a higher number of ICs were contaminated by electrical cardiac interference in G2 and G3. These results demonstrate that our approach, exploiting the time and frequency information provided by the real ECG signals, was effective in separating the artefactual signals due to the electrical and pulsatile activity of the neonatal heart in a small number of ICs. Moreover, the better separation of the cardiac-related interferences in few ICs also yielded an improvement in the automated classification of the ICs. Of the groups of seizure-free EEG segments, the two signal groups exploiting the additional nECG (i.e., G1 with 21 ICs and G2 with 20 ICs) had the best automated classification performance for the ICs containing the electrical cardiac artefacts (ECC): 20 out of 21 artefactual ICs were correctly classified for G1, and 20 out of 20 artefactual ICs were correctly classified for G2. Similarly, the automated classification of the ICs containing the pulsatile activity of the neonatal heart achieved the best results when nAPS was included in the pool of input signals (G1), outperforming the automated classification obtained with the other signal groups (G2 and G3) (see Table 2). These results were further confirmed by the automated classification of the electrical cardiac and pulsatile ICs (ECC and PCC), which were separated from the EEG signals containing seizures (G1s) by exploiting both nECG and nAPS: 17 out of 20 ICs containing electrical cardiac artefacts and all ICs containing pulsatile interference were correctly classified.

As compared with the classification performed by the experienced investigators, the automated classification of the ICs achieved extremely good results when using both nECG and nAPS together with the EEG signals: for both G1 and G1s, the classification accuracy was 0.99, the false omission rate was 0.01, and the sensitivity was 0.93. These values decreased when only the nECG and the EEG signals or only the EEG signals were used, demonstrating the usefulness of the time and frequency information provided by the real ECG signals to achieve an effective separation, and consequently an excellent automated classification of the artefactual ICs. Other authors developing methods to detect the electrical cardiac and/or pulsatile interference in neonatal EEG recordings did not assess the performance of their classifiers in terms of statistical measures such as accuracy, sensitivity and false omission rate [13,35,39,41,51]. Therefore, a direct comparison of the performance of our automated classification method with the performance of other similar methods was not possible. However, of what could be estimated from the reported validation of existing classifiers for neonatal EEG applications, it seems that the performance of our automated classification of the ICs containing electrical cardiac interference was comparable—and sometimes better—than the classification performance of previous methods, and that the classification of ICs containing pulsatile interference performed with our approach outperformed other methods [13,39,41].

It is worth noting that, with respect to other authors [13,35,39,41,51], we not only detected the cardiac-related interference but also removed it and verified whether the neonatal brain signals were preserved afterwards. To this aim, we evaluated the variation in the neonatal EEG signal power (SPV) before and after removal of the ICs containing electrical cardiac and pulsatile interference. The small percent variation in the neonatal EEG signal power observed for all seizure-free EEG signal groups (median SPV < 13%) demonstrated that the use of nECG and nAPS, by improving the source signal separation better preserved the EEG signals in the non-artefactual ICs. Similar results were obtained when the percent variation in the neonatal EEG signal power was calculated for the EEG segments extracted from EEG recordings including seizures (median SPV = 8.4%), confirming the effectiveness of our method in removing artefactual signal components without altering true brain signals.

We further calculated the percentage variation in the power of the EEG signals at the R peaks (varRPP), which was reduced by more than 60% and by about 80% when the source signal separation was performed using the nECG on the EEG signals without and with seizures, respectively. The larger signal power reduction in the EEG signals containing seizures is most likely due to the higher R peak amplitude in this type of recording. Please note that the value of varRPP decreased to about 45% for the signal group containing only EEG signals. On the other hand, given that the pulse signal has the same frequency content as the ECG, we observed that the PSD amplitude at the heart rate frequency of all reconstructed EEG signals (varPSDhr) was reduced by 90% when the nAPS was added to the pool of EEG signals for the SOBI decomposition. A similar result was obtained with the EEG signals containing seizures (G1s), and the significant difference with the results obtained with the seizures-free EEG signals (G1) is most probably due to the higher variability of the varPSDhr values across the EEG signals containing seizures, which, in turn, is related to the higher number of ICs containing both electrical cardiac and pulsatile interference. The results for varRPP and varPSDhr demonstrate that adding the nECG and nAPS to the pool of EEG signals improves the separation of the source signals, and hence effectively reduces the amount of signal power related to the electrical activity of the heart and the pulsatile activity of the blood vessels that are still present in the reconstructed neonatal EEG signals.

The overall performance of our proposed method was, therefore, very good for both the EEG recordings with no annotations for clinically relevant conditions and the EEG recordings containing seizures. The results obtained for the EEG recordings with seizures are particularly valuable because this type of EEG signal is typically affected by a higher level of noise. However, some limitations should be addressed in future studies. From an applicative perspective, although in the case of neonates hospitalized in the NICU, the ECG is always recorded together with the EEG, our method could be more widely used if it would not require the simultaneously recording of the ECG signals. This issue could be addressed by developing a model of the ECG and pulse signals—based on the temporal and frequency features of the real ECG and pulse signals in infants—which could be used as an input to the BSS decomposition. To provide more generalizable results, our method should also be validated in neonatal datasets with less than 19 EEG signals because a low number of EEG channels is often used in clinical recordings of the neonatal brain activity. Although SOBI was proved to be ineffective for EEG datasets with a low number of signals, the improvement achieved with our method is promising and could lead to an effective decomposition with few input channels.

## 5. Conclusions

We proposed a method the separate and remove electrical cardiac and pulsatile interference from neonatal EEG recordings that exploits the temporal and frequency features of the real ECG simultaneously recorded in neonates hospitalized in the NICU. Our new approach, tested on both seizure-free EEG recordings and EEG recordings containing seizures, seemed to outperform the existing methods to detect these types of interference and—with respect to existing methods—was able to remove those interferences while preserving true brain activity in the reconstructed neonatal EEG signals. To increase the applicability of our method, future studies should attempt to make it independent of the simultaneous recording of ECG signals and test it in EEG recordings with a lower number of channels.

## Figures and Tables

**Table 1:** The descriptive statistics of the number of ICs classified as ECC or PCC is shown for the different signal groups G1, G2 and G3 (i.e., for the decomposition levels to 21 ICs, 20 ICs and 19 ICs, respectively). From left to right: type of artefactual IC, decomposition level, median, 95th confidence interval, Chi-square and the *p*-value of the Friedmann test, type of paired post-hoc comparisons, Z value, *p*-value and effect size of the Wilcoxon test.

Artefactual IC	Decomposition Level	Median	95th CI	𝜒^2^	*p*	Comparison	Z	*p_w_*	Effect Size
ECC	21 ICs	1.0	0.00			21 ICs vs. 20 ICs	0.00	1.00	0.00
	20 ICs	1.0	0.00	14.70	<0.001	21 ICs vs. 19 ICs	3.74	<0.001	0.59
	19 ICs	0.0	0.21			20 ICs vs. 19 ICs	3.74	<0.001	0.59
PCC	21 ICs	2.00	0.25			21 ICs vs. 20 ICs	3.61	<0.05	0.57
	20 ICs	1.00	0.17	19.43	<0.001	21 ICs vs. 19 ICs	4.24	<0.001	0.67
	19 ICs	1.00	0.27			20 ICs vs. 19 ICs	2.24	<0.05	0.35

**Table 2:** Outcome of the validation of the automated classification of artefactual ICs as ECC and PCC as compared with the classification performed by the experienced investigators. Results are shown separately for each signal group (G1, 21 ICs; G2, 20 ICs; G3, 19 ICs). In the ECC and PCC columns, the number of ICs classified by the expert investigators is reported first (outside the parenthesis), whereas the number of ICs automatically classified is reported within the parenthesis.

Signal Group	Dec. level	No. of ICs	ECC	PCC	TP	TN	FP	FN	Acc	FOR	Sens
G1	21 ICs	420	21(20)	36(34)	53	362	1	4	0.99	0.01	0.93
G2	20 ICs	400	20(20)	28(21)	41	352	0	7	0.98	0.02	0.85
G3	19 ICs	380	20(6)	27(16)	22	333	0	25	0.93	0.07	0.47

**Table 3:** Outcome of the validation of the automated classification of artefactual ICs as ECC and PCC, as compared with the classification performed by the experienced investigators. Results are shown for the signal group G1s, including EEG segments containing seizures, and for the group G1 on seizures-free EEG segments, both decomposed in 21 ICs. In the ECC and PCC columns, the number of ICs classified by the expert investigators is reported first (outside the parenthesis), whereas the number of automatically classified ICs is reported within the parenthesis.

Signal Group	Dec. level	No. ofICs	ECC	PCC	TP	TN	FP	FN	Acc	FOR	Sens
G1s	21 ICs	420	20(17)	29(29)	46	371	0	3	0.993	0.008	0.939
G1	21 ICs	420	21(20)	36(34)	53	362	1	4	0.988	0.011	0.930

## Data Availability

The EEG dataset used in this study was obtained from a freely available online repository (Zenodo, https://zenodo.org/record/2547147#.YUr9by8QNUM. accessed on 5 June 2018).
